# Comparison of thalamic atlases and segmentation techniques in defining motor and sensory nuclei for deep brain stimulation targeting in essential tremor

**DOI:** 10.1016/j.nicl.2025.103887

**Published:** 2025-09-27

**Authors:** Mathew E. Zilberman, Kelvin L. Chou, Parag G. Patil, Karlo A. Malaga

**Affiliations:** a Department of Biomedical Engineering, Bucknell University, Lewisburg, PA, USA; b Department of Neurology, University of Michigan, Ann Arbor, MI, USA; c Department of Neurosurgery, University of Michigan, Ann Arbor, MI, USA; d Department of Biomedical Engineering, University of Michigan, Ann Arbor, MI, USA

**Keywords:** Deep brain stimulation, Essential tremor, Thalamus, Diffusion tensor imaging, Thalamic segmentation, Thalamic atlas, Volume of tissue activation

## Abstract

**Introduction:**

Many thalamic atlases are available for deep brain stimulation (DBS) applications, but their usage and clinical validation vary. This study investigated the effectiveness of six atlases in DBS targeting for essential tremor (ET) through structural differences, impact of two thalamic segmentation methods, and correspondence with clinical outcomes via individualized tissue activation modeling.

**Methods:**

22 ET patients with unilateral VIM DBS were retrospectively analyzed. Volume of tissue activation (VTA) models were linked to tremor reduction (n = 22) and sustained paresthesia (n = 32). Six atlases were co-registered for pairwise comparison. Patient thalami were segmented using atlas-based segmentation (ABS) and diffusion tensor imaging-based segmentation (DTIBS). Geometric properties and VTA overlap with motor and sensory regions were statistically assessed.

**Results:**

Atlas comparisons revealed significant differences in motor and sensory subnuclei delineation (p < 0.001). ABS generally produced larger motor and smaller sensory volumes than DTIBS, with both showing significant geometric variability. For therapeutic VTAs, ABS consistently showed greater motor activation across all atlases, while DTIBS demonstrated this in only half. Sensory activation was more often greater for paresthesia than therapeutic VTAs using ABS. The Jakab atlas showed the strongest correspondence with clinical outcomes using both segmentation methods.

**Conclusion:**

Atlas choice and segmentation method can potentially influence DBS targeting accuracy. A segmentation approach that performs well with one atlas may not generalize to others, underscoring the need for clinical validation to evaluate applicability in surgical planning. DTIBS may enable more individualized targeting, but requires further refinement to consistently outperform traditional methods.

## Highlights

• Compared clinical consistency of 6 thalamic atlases and 2 segmentation methods.
• Motor and sensory thalamic subnuclei divisions vary significantly across atlases.
• Atlas-based segmentation corresponded better with clinical outcomes than DTI-based.
• DTI-based segmentation was more sensitive to atlas choice.
• The Jakab atlas was the most clinically applicable atlas examined.

## 1 Introduction

Thalamic deep brain stimulation (DBS) is a proven treatment for both Parkinsonian and essential tremor (ET) (Baizabal-Carvallo et al., 2014, Benabid et al., 1991, Kremer et al., 2021). The thalamus can be divided functionally and anatomically into smaller subnuclei, with the ventral intermediate (VIM) (Benabid et al., 1996, Flora et al., 2010) or the ventral part of the ventral lateral posterior (Schaltenbrand and Wahren, 1977) nucleus as the established stimulation target for DBS used to treat ET. Since most clinical magnetic resonance imaging (MRI) does not have the resolution to allow for observable partitioning of thalamic subnuclei (Chung et al., 2020, Kanowski et al., 2014, Mang et al., 2012, McDannold et al., 2020, Sammartino et al., 2016, Williams et al., 2024), neurosurgeons most often rely on indirect targeting techniques, such as stereotactic atlases (Behrens et al., 2003, Chandra et al., 2022, Iorio-Morin et al., 2020) together with intraoperative physiological testing, to locate relevant motor subnuclei for stimulation.

However, there are a plethora of thalamic atlases available for DBS applications, each with widely contrasting delineations (Neudorfer et al., 2024) and nomenclature (Mai and Majtanik, 2019) of subnuclei involved in motor and sensory signaling. These differences arise from individual topographical variation, the influence of age and disease, and the different weights placed on cytoarchitectural anatomy and functional connectivity (Mai and Majtanik, 2019). The usage and degree of clinical validation vary across atlases, raising a practical challenge of deciding which atlas to use for surgical planning purposes. Traditional atlases represent average patient anatomy, and individual patients may deviate from these generalized references. In more data-driven approaches, diffusion tensor imaging (DTI) is used to segment the thalamus in an individualized manner by forming clusters of voxels with similar tissue properties (Battistella et al., 2017, Malaga et al., 2022, Wiegell et al., 2003). Atlases still influence this process though, as the number of subnuclei in an atlas can dictate how many clusters are generated. Consequently, segmentation methods may be dependent on the chosen atlas, and those optimized for one atlas may not generalize well to others.

The objective of this research study was to analyze the comparative effectiveness of available thalamic atlases in targeting motor subnuclei. Specifically, this study investigated whether there is an appreciable difference among atlases, how thalamic segmentation methods impact these differences, and which atlases and segmentation techniques ultimately best correspond with clinical outcomes based on individualized tissue activation modeling (Malaga et al., 2021). In doing so, this work provides insight into the clinical applicability of various atlases and segmentation methods in thalamic DBS for ET.

## 2 Methods

### 2.1 Clinical data

The patient data used in this retrospective study was sourced from a clinical database at the University of Michigan (Malaga et al., 2022). Twenty-two patients (sex: 14 male, 8 female; age at surgery: 66.9 ± 9.1 years (mean ± SD); age at diagnosis: 40.0 ± 21.3 years; disease duration: 26.8 ± 20.7 years; handedness: 3 left, 19 right) with medically intractable ET were previously treated with unilateral VIM DBS (hemisphere: 19 left, 3 right) for upper extremity tremor in the contralateral limb. All patients had medical imaging and clinical assessment data available, and did not have MRI structural brain abnormalities, non-monopolar stimulation, or comorbid neuropsychiatric conditions. In the surgeries performed, preliminary targeting used standard indirect stereotactic coordinates relative to anatomical landmarks: 11.5 mm lateral to the border between the thalamus and the third ventricle, 3 mm anterior to the posterior commissure, and aligned with the intercommissural plane. Intraoperative microelectrode macrocontact stimulation subsequently refined targeting, and a quadripolar DBS lead (model 3387, Medtronic plc, Minneapolis, MN, USA) was implanted. A monopolar review was then performed intraoperatively and six weeks postoperatively to optimize stimulation settings (Supplementary Table 1). To minimize potential observer bias, the review was done by the same movement disorder neurologist, who was blinded to the thalamic segmentation and tissue activation modeling, at each electrode contact following standard procedures. Tremor improvement, defined by disappearance or reduction of tremor, was recorded during monopolar review for all patients.

Volume of tissue activation (VTA) models were obtained from previous work (Malaga et al., 2022). These models were derived from preoperative MRI (Achieva 3 T, Philips Healthcare, Andover, MA, USA), postoperative computerized tomography (CT) (Patil et al., 2012), and monopolar stimulation settings that yielded both therapeutic and side-effect outcomes. Patients with tremor reduction outcomes (n = 22) were included in the therapeutic analysis, with each patient having a single active electrode contact, totaling 22 tremor reduction VTAs. Therapeutic contacts were programmed with an amplitude of 1.7 ± 0.5 V (n = 18) or 1.9 ± 0.5 mA (n = 4), a frequency of 130 Hz, and a pulse width of 60 μs. Therapeutic VTAs were associated with each patient’s clinically optimized contact and settings from DBS programming. The degree of tremor reduction was not specifically investigated due to the unavailability of pre- and post-operative tremor scores. A subset of patients with sustained paresthesia outcomes (n = 12) were included in the side effect analysis, with ten patients having multiple individual electrode contacts (range: 2–4 contacts), totaling 32 sustained paresthesia VTAs. Sustained paresthesia contacts were programmed with an amplitude of 2.8 ± 1.3 V (n = 23) or 2.5 ± 0.9 mA (n = 9), a frequency of 130 Hz, and a pulse width of 60 μs. Sustained paresthesia VTAs were associated with contacts and settings from programming that elicited paresthesia lasting more than one minute.

Preoperative DTI that was captured using single-shot echo-planar imaging (number of gradient directions: 15; b-value: 800 s/mm^2^; voxel size: 1 × 1 × 2 mm^3^; field-of-view: 224 × 224 mm^2^; reduction factor: 2) was similarly obtained from previous work (Malaga et al., 2022). All imaging data were previously co-registered, with MRI rigidly transformed to Talairach space along the intercommissural and midsagittal planes (Talairach and Tournoux, 1988) using Analyze (Analyze 12.0, AnalyzeDirect, Inc., Overland Park, KS, USA), and DTI and CT aligned to the Talairach-oriented MRI via semiautomatic linear registration. Anatomical models of the patient thalami, which had been created by manually tracing the borders of the thalamus from coronal MRI slices, were obtained from previous analysis (Malaga et al., 2022). Thalamic borders were defined by the third ventricle and posterior limb of the internal capsule in the axial plane, and by the lateral ventricle and subthalamic nucleus in the sagittal plane (Power et al., 2015). The Institutional Review Board at Bucknell University (2122–056) and at the University of Michigan (HUM00021058) approved the use of this data for the study. Written informed consent was obtained from all patients.

### 2.2 Thalamic atlas acquisition, classification, and processing

**Fig. 1:**
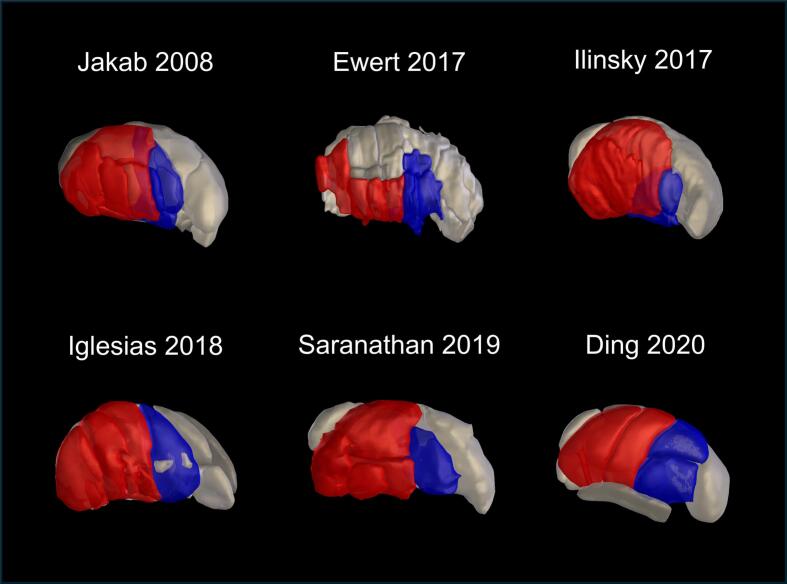
The six thalamic atlases examined. Qualitative differences in motor (red) and sensory (blue) subnuclei among the atlases are shown. (For interpretation of the references to colour in this figure legend, the reader is referred to the web version of this article.)

Six prominent thalamic atlases (Ding, Ewert, Iglesias, Ilinsky, Jakab, and Saranathan) (Chakravarty et al., 2006, Ding et al., 2016, Iglesias et al., 2018, Ilinsky et al., 2018, Jakab et al., 2012, Morel, 2007, Saranathan et al., 2021) with different motor-sensory boundaries (Fig. 1) and subnuclei counts (Table 1) were obtained from Lead-DBS (Horn and Kühn, 2015), a toolbox that enables DBS electrode visualization based on patient MRI and CT imaging, as MATLAB MAT-files. All atlases were standardized in Lead-DBS and represented as MNI (Montreal Neurological Institute) 2009b nonlinear asymmetric T2 templates with a resolution of 0.5 × 0.5 × 0.5 mm^3^. The ventral anterior, ventral lateral, and ventral intermediate thalamic subnuclei were classified as motor thalamus and the ventral posterior lateral and ventral posterior medial subnuclei as sensory thalamus (Mai and Majtanik, 2019, Neudorfer et al., 2024, Schaltenbrand and Wahren, 1977). The motor and sensory regions were defined as the union of their respective subnuclei (Supplementary Table 2) (Malaga et al., 2022). This was done to facilitate comparisons across atlases. Because the anatomical terminology and delineations varied between atlases, direct one-to-one comparisons of individual subnuclei were unfeasible.

**Table 1:** Analyzed thalamic atlases with motor and sensory subnuclei breakdowns and abbreviations.

Atlas*	No. of Total Subnuclei	No. of Motor Subnuclei	No. of Sensory Subnuclei	Abbreviation
Jakab 2008	32	6	4	Jak08
Ewert 2017	46	5	9	Ewt17
Ilinsky 2017	15	3	2	Iln17
Iglesias 2018	23	4	1	Igl18
Saranathan 2019	10	4	1	Sar19
Ding 2020	13	2	3	Dng20

* Atlas names from Lead-DBS.

In all atlases, the subnuclei belonging to the thalamus were isolated (excluding the medial and lateral geniculate nuclei) and artifact points were removed using thresholding. For the inter-atlas spatial analysis, the left thalamus of each atlas was isolated and co-registered with that of every other atlas using an iterative closest point (ICP) algorithm. All atlases contained distinct bilateral thalami, but the left thalamus was chosen for the inter-atlas analysis because 19 of 22 patients had implants in their left hemisphere. The Dice coefficient (DC) was used to determine the similarity of the entire thalamus, motor subnuclei, and sensory subnuclei between atlases. This metric ranges from 0 to 1, where 1 indicates identical geometries. The DC was calculated as:

(1) $$\mathrm{DC}=\frac{2\left|V_{\mathrm{Fixed}}\cap V_{\mathrm{Moving}}\right|}{\left|V_{\mathrm{Fixed}}\right|+\left|V_{\mathrm{Moving}}\right|}$$

where $V_{\mathrm{Moving}}$ indicates the volume of the atlas that was registered to $V_{\mathrm{Fixed}}$, the volume of the fixed atlas.

**Fig. 2:**
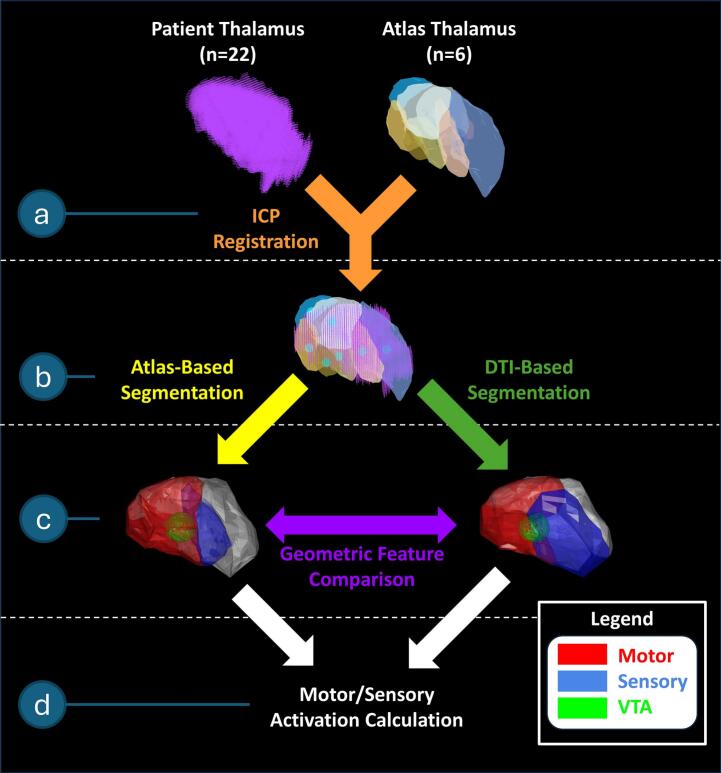
Complete segmentation and analysis workflow. For each patient and atlas pair, the segmentation approach involved (a) performing an ICP registration, (b) segmenting using both ABS and DTIBS techniques, (c) conducting a geometric feature comparison, and (d) calculating motor and sensory activation.

**Fig. 3:**
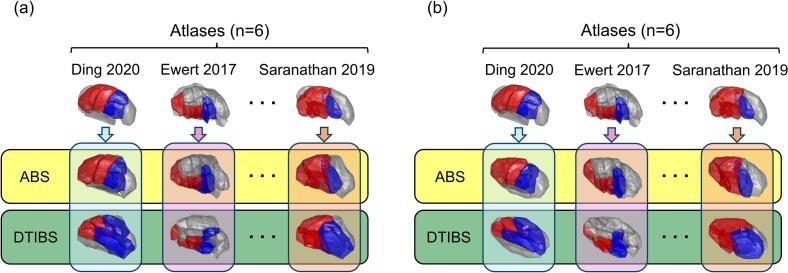
Comparison of segmentation approaches. An example of ABS and DTIBS techniques used with three atlases to segment the motor (red) and sensory (blue) subnuclei is shown for two exemplary patients (a) and (b). (For interpretation of the references to colour in this figure legend, the reader is referred to the web version of this article.)

In the main segmentation workflow, the thalamus matching the side of the patient’s implant was chosen for all atlases and co-registered with each patient’s thalamus using the ICP algorithm (Fig. 2a). The registered atlases were then used to segment the patient thalami using two methods: atlas-based segmentation (ABS) or DTI-based segmentation (DTIBS) (Fig. 2b). This was performed across all atlases (Fig. 3).

Geometric features (volume and centroid distance, relative to the thalamus centroid) were calculated for both motor and sensory subnuclei as segmented by ABS or DTIBS (Fig. 2c). Thalami segmented by ABS and DTIBS were clinically validated using VTA modeling (Fig. 2d), and this process was performed for all patients. The entire segmentation and analysis workflow was executed in each patient’s native space using MATLAB (R2024a, The MathWorks, Inc., Natick, MA, USA).

### 2.3 Thalamic segmentation techniques

#### 2.3.1 Atlas-based segmentation

In the traditional ABS method, patient thalamic voxels were categorized by the subnucleus they resided in or the closest subnucleus boundary if they did not reside in any subnuclei. Namely, divisions between subnuclei were prescriptive anatomical boundaries applied to the patient thalamus by the registered atlases. ABS was performed for each patient and atlas combination (n = 132).

#### 2.3.2 DTI-based segmentation

In contrast to ABS, DTIBS utilized a more data-driven approach whereby the atlases informed the initial clustering conditions, but DTI-derived parameters guided patient-specific segmentation. The subnuclei centroids of the registered atlases served as seed points for a *k*-means clustering algorithm. Thus, the number of final clusters equaled the number of subnuclei in the atlas, which ranged from k = 10 to k = 46 clusters (Table 1). The distance metric ($d_{\mathrm{jk}}$), based on a previous study (Malaga et al., 2022), was a linear combination of the Mahalanobis voxel distance (Wiegell et al., 2003) and the Hybrid tensor distance (García et al., 2012):

(2) $$d_{\mathrm{jk}}={\|x_{j}-{\overset{¯}{x}}_{k}\|}_{W_{k}}+\alpha \left\|K\left(D_{j}\right)-K\left({\overset{¯}{D}}_{k}\right)\right\|+\left(1-\alpha \right){\|U\left(D_{j}\right)-U\left({\overset{¯}{D}}_{k}\right)\|}_{F}$$

where $x_{j}$ is the coordinate of voxel j, ${\overset{¯}{x}}_{k}$ is the centroid coordinate of subnucleus k, $W_{k}$ is the covariance matrix of the voxels in subnucleus k, $D_{j}$ is the diffusion tensor of voxel j, and ${\overset{¯}{D}}_{k}$ is the mean diffusion tensor of subnuclei k. K is a feature vector containing three tensor invariants that describe the shape of the diffusion tensor, U is a tensor describing the primary orientation of the diffusion tensor, and α is a weighting factor controlling the importance of tensor shape and orientation (α = 0.5) (García et al., 2012). The Mahalanobis distance accounts for spatial difference, while the Hybrid distance accounts for tensor dissimilarity. The *k*-means algorithm was iterated until the convergence criteria were satisfied: the movement of each centroid was less than 0.1 mm (Wiegell et al., 2003). The sensitivity of the algorithm to the cluster initialization was assessed across all patient and atlas combinations by performing 100 segmentations, each time randomly displacing the coordinates of each subnucleus centroid within a 2-mm radius. DTIBS was done for each patient-atlas pair (n = 132).

### 2.4 Tissue activation modeling and calculation

Finite element analysis using CT-derived DBS lead position, DTI-derived anisotropic tissue conductivity, and clinical stimulation settings was previously performed for all patients in COMSOL (COMSOL Multiphysics 5.2, COMSOL, Inc., Burlington, MA, USA) to model the VTA. The tissue surrounding the active contact with an electric field norm greater than 0.2 V/mm defined the VTA boundary. The overlap between the VTA and the motor and sensory regions was calculated as:

(3) $$\mathrm{VTA}{\mathrm{Region}}_{\mathrm{Motor}O\mathrm{rSensory}}Overlap=\frac{\mathrm{VTA}\cap {\mathrm{Region}}_{\mathrm{Motor}O\mathrm{rSensory}}}{\mathrm{VTA}}\times 100$$

and was used to quantify motor and sensory activation for therapeutic (n = 22) and paresthesia (n = 32) VTAs.

### 2.5 Statistical analysis

Subnuclei centroids were calculated by averaging all voxel points of the subnucleus. Paired, two-sided Wilcoxon signed-rank tests were used to determine whether a significant difference existed between the inter-atlas overlap of the entire thalamus and that of both motor and sensory subnuclei. Paired, two-sided Wilcoxon signed-rank tests were also used to determine whether the geometric features differed significantly between the motor and sensory regions of patient thalami segmented with ABS and DTIBS, as well as whether the VTA-motor and -sensory overlap differed significantly between tremor reduction and sustained paresthesia outcomes. As ten patients had one therapeutic VTA and two to four paresthesia VTAs, the VTA-sensory overlap values for therapeutic VTAs were duplicated in the paresthesia correspondence analysis to enable pairwise comparison. All statistical analysis was performed using MATLAB. Statistical significance was determined by a p-value less than 0.05, which was adjusted for the six atlas comparisons using the Bonferroni-Holm method.

## 3 Results

### 3.1 Spatial overlap of thalamic atlases

Whole thalamus overlap was high across all atlas pairs, with DC of at least 0.70 (Table 2a). For the motor subnuclei, the Ewert atlas showed the lowest average overlap with the corresponding regions in the other five atlases (mean DC = 0.35), while the Jakab atlas had the highest (mean DC = 0.67) (Table 2b). For the sensory subnuclei, the Ilinsky atlas showed the lowest average overlap with the corresponding regions in the other atlases (mean DC = 0.44), while the Ewert atlas had the highest (mean DC = 0.58) (Table 2c). Overall, the overlap between motor subnuclei across all non-duplicative atlas pairs was significantly lower than that of the whole thalamus (p < 0.001); the same was true for the sensory subnuclei (p < 0.001). There was no significant difference between the DC of the motor and sensory subnuclei.

**Table 2a:** DC of whole thalamus across all atlas pairs.*

		Moving Atlas					
		Dng20	Ewt17	Igl18	Iln17	Jak08	Sar19
**Fixed Atlas**	**Dng20**	1	0.85	0.82	0.76	0.81	0.84
	**Ewt17**	0.84	1	0.84	0.79	0.85	0.82
	**Igl18**	0.85	0.86	1	0.87	0.92	0.80
	**Iln17**	0.79	0.81	0.89	1	0.89	0.73
	**Jak08**	0.82	0.84	0.90	0.86	1	0.78
	**Sar19**	0.85	0.85	0.79	0.70	0.79	1

**Table 2b:** DC of motor subnuclei across all atlas pairs.*

		Moving Atlas					
		Dng20	Ewt17	Igl18	Iln17	Jak08	Sar19
**Fixed Atlas**	**Dng20**	1	0.26	0.61	0.64	0.69	0.74
	**Ewt17**	0.25	1	0.38	0.27	0.45	0.42
	**Igl18**	0.64	0.35	1	0.81	0.78	0.72
	**Iln17**	0.69	0.28	0.77	1	0.72	0.71
	**Jak08**	0.70	0.46	0.76	0.70	1	0.75
	**Sar19**	0.77	0.42	0.69	0.70	0.74	1

**Table 2c:** DC of sensory subnuclei across all atlas pairs.*

		Moving Atlas					
		Dng20	Ewt17	Igl18	Iln17	Jak08	Sar19
**Fixed Atlas**	**Dng20**	1	0.62	0.53	0.47	0.58	0.45
	**Ewt17**	0.63	1	0.48	0.50	0.68	0.60
	**Igl18**	0.58	0.52	1	0.52	0.56	0.41
	**Iln17**	0.43	0.53	0.41	1	0.43	0.26
	**Jak08**	0.58	0.65	0.50	0.42	1	0.56
	**Sar19**	0.48	0.60	0.36	0.39	0.57	1

* Moving atlas registered to fixed atlas.

### 3.2 Subnuclei geometry comparison between ABS and DTIBS techniques

**Fig. 4:**
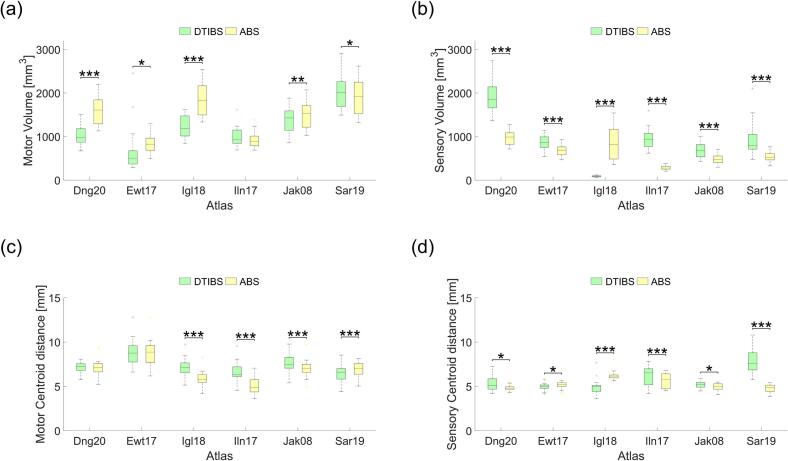
Geometric comparison between segmentation techniques. Total volume of (a) motor and (b) sensory subnuclei for DTIBS (green) and ABS (yellow). Centroid distance (relative to thalamus centroid) of (c) motor and (d) sensory subnuclei for DTIBS and ABS. * p < 0.05, ** p < 0.01, *** p < 0.001; paired, two-sided Wilcoxon signed-rank test; n = 22. (For interpretation of the references to colour in this figure legend, the reader is referred to the web version of this article.)

The volume and centroid distance (relative to the thalamus centroid) of both motor and sensory subnuclei varied between segmentation methods and across atlases. In four of the six atlases, the volume of motor thalamus was significantly larger when segmented using ABS compared to DTIBS, with the opposite trend observed in the Saranathan atlas (Fig. 4a). Sensory thalamus volume was significantly smaller with ABS than with DTIBS in five of six atlases, with the Iglesias atlas showing the opposite trend (Fig. 4b). Across all atlases, ABS yielded average motor and sensory volumes of 1423 ± 515 mm^3^ and 623 ± 296 mm^3^, respectively, while DTIBS yielded volumes of 1211 ± 535 mm^3^ and 904 ± 595 mm^3^. When aggregated across all patients and atlases, ABS yielded a significantly larger motor volume (p < 0.001, n = 132) and a significantly smaller sensory volume (p < 0.001, n = 132) than DTIBS.

Motor thalamus centroid distance was significantly different between segmentation methods in four of six atlases. In three of these four atlases, DTIBS yielded a larger motor centroid distance than ABS (Fig. 4c). In contrast, sensory thalamus centroid distance differed significantly between ABS and DTIBS in all six atlases, with DTIBS yielding a greater centroid distance in four of them (Fig. 4d). Across all atlases, ABS yielded average motor and sensory centroid distances of 6.76 ± 1.53 mm and 5.24 ± 0.72 mm, respectively, while DTIBS yielded centroid distances of 7.26 ± 1.29 mm and 5.73 ± 1.31 mm. When aggregated across all patients and atlases, DTIBS yielded significantly greater centroid distances than ABS for both motor (p < 0.001, n = 132) and sensory (p = 0.0018, n = 132) subnuclei.

### 3.3 Clinical correspondence with tremor reduction

**Fig. 5:**
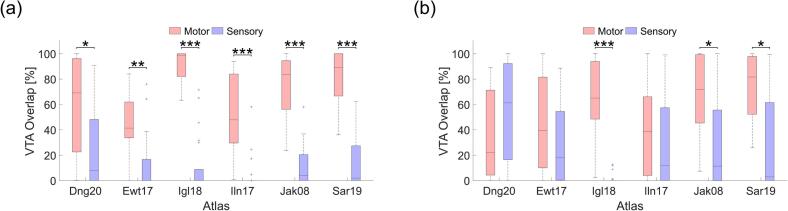
Tremor reduction correspondence across atlases. VTA-motor (red) and -sensory (blue) overlap for therapeutic outcomes using (a) ABS and (b) DTIBS. * p < 0.05, ** p < 0.01, *** p < 0.001; paired, two-sided Wilcoxon signed-rank test; n = 22. (For interpretation of the references to colour in this figure legend, the reader is referred to the web version of this article.)

For VTAs associated with therapeutic outcomes, ABS yielded average VTA-motor overlaps ranging from 45 % ± 26 % (Ewert atlas) to 91 % ± 11 % (Iglesias atlas), and VTA-sensory overlaps ranging from 5 % ± 13 % (Ilinsky atlas) to 25 % ± 33 % (Ding atlas). In comparison, DTIBS yielded average VTA-motor overlaps ranging from 34 % ± 34 % (Ding atlas) to 74 % ± 26 % (Saranathan atlas), and VTA-sensory overlaps ranging from 2 % ± 4 % (Iglesias atlas) to 55 % ± 39 % (Ding atlas). ABS showed a significantly greater VTA-motor overlap than VTA-sensory overlap in all six atlases (Fig. 5a), while DTIBS had this in only three of six atlases (Fig. 5b). Across atlases, the interquartile range of the VTA-motor and -sensory overlap was generally wider with DTIBS than with ABS. Notably, patient thalami segmentation with the Ilinsky atlas using ABS and with the Iglesias atlas using DTIBS showed VTA-sensory overlap values near zero.

### 3.4 Clinical correspondence with sustained paresthesia

**Fig. 6:**
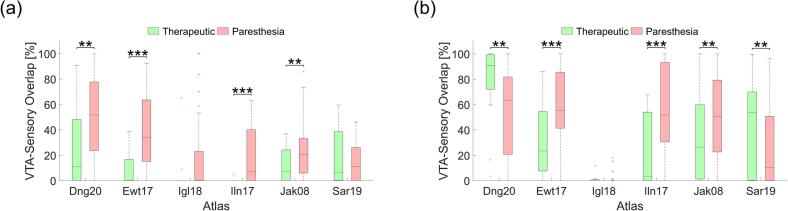
Sustained paresthesia correspondence across atlases. VTA-sensory overlap for therapeutic (green) and paresthesia (red) outcomes using (a) ABS and (b) DTIBS. * p < 0.05, ** p < 0.01, *** p < 0.001; paired, two-sided Wilcoxon signed-rank test; n = 32. (For interpretation of the references to colour in this figure legend, the reader is referred to the web version of this article.)

Using the ABS technique, average VTA-sensory overlaps for tremor reduction-associated VTAs ranged from 1 % ± 2 % (Ilinsky atlas) to 25 % ± 32 % (Ding atlas), while overlaps for paresthesia-associated VTAs ranged from 14 % ± 15 % (Saranathan atlas) to 50 % ± 31 % (Ding atlas). For DTIBS, average VTA-sensory overlaps for therapeutic VTAs ranged from 1 % ± 3 % (Iglesias atlas) to 77 % ± 31 % (Ding atlas), and overlaps for paresthesia VTAs ranged from 1 % ± 4 % (Iglesias atlas) to 59 % ± 29 % (Ewert atlas). VTA-sensory overlap was significantly greater for paresthesia VTAs compared to therapeutic VTAs in four of the six atlases using ABS (Fig. 6a). In contrast, DTIBS yielded greater VTA-sensory overlap for paresthesia VTAs in three of six atlases, but greater overlap for therapeutic VTAs in two of six atlases (Fig. 6b). Notably, the VTA-sensory overlap values for the Iglesias atlas when using DTIBS were near zero for both paresthesia and therapeutic VTAs. When using ABS, both the Iglesias and Ilinsky atlases showed near-zero VTA-sensory overlap values for only therapeutic VTAs.

## 4 Discussion

The aim of this study was to determine the influence of atlas choice on defining the motor and sensory regions of the thalamus using two distinct segmentation approaches. While previous studies have compared segmentation techniques (Ewert et al., 2019, Williams et al., 2024) and thalamic atlases (Mai and Majtanik, 2019), to the authors’ knowledge, this study marks the first comprehensive investigation into the relationships among thalamic segmentation, VTA location, and DBS clinical outcome, across multiple atlases.

### 4.1 Atlas differences

Registering entire thalamic atlases onto one another yielded much larger whole-thalamus DC than individual motor and sensory DC, indicating that the locations of motor and sensory regions, in addition to the number of motor and sensory subnuclei, vary significantly among atlases relative to the whole thalamus. This finding aligns with these atlases having heterogeneous motor thalamus parcellations (Neudorfer et al., 2024). Potential reasons for inconsistency between the thalamic atlases included in this study are varying subject age, different atlas reconstruction methodologies, and bias towards functional connectivity or anatomical structure (Mai and Majtanik, 2019). The Jakab, Iglesias, and Saranathan atlases were derived from two, six, and nine subjects with average ages of 65 ± 6 years, 81 ± 16 years, and 32 ± 5 years, respectively (Iglesias et al., 2018, Morel, 2007, Saranathan et al., 2021), whereas the other three atlases were from one subject (age: 34 years for the Ding atlas (Ding et al., 2016), unreported for the Ewert and Ilinsky atlases). All atlases were developed using anatomical methods, mainly histology and MRI; the Saranathan atlas solely used 7 T MRI (Saranathan et al., 2021). However, the delineations of the motor subnuclei in the Jakab atlas are based on the labeling proposed by Hirai and Jones (Hirai and Jones, 1989), which emphasized functional connectivity over cytoarchitecture. Additionally, all atlases were derived from brains from subjects without neurological conditions, which potentially reduces their applicability for patients with ET or other neurological disorders.

### 4.2 Segmentation differences

Across all atlases, the sensory subnuclei had significantly different centroid distance and volume between ABS and DTIBS. In contrast, the motor subnuclei had slightly more consistent geometric features between segmentation approaches and across atlases. The greater centroid distance associated with DTIBS for both motor and sensory subnuclei suggested its increased variability compared to ABS. Using DTIBS, stability was found to be atlas-dependent: atlases with a larger number of subnuclei, and thus more seed points, produced more stable clusters.

While the Ding and Ewert atlases had similar motor subnuclei centroid distance between ABS and DTIBS, they both had different motor volume between approaches. In contrast, the Ilinsky atlas’ relatively consistent motor volume between segmentation techniques coincided with a significant difference in motor centroid distance. Overall, all atlases had significantly different sensory volume and sensory centroid distance between ABS and DTIBS, and at least one significantly different motor geometric feature. These findings indicated that no atlas was immune to the variability stemming from the choice of segmentation approach. Thus, choosing a segmentation technique based on either patient-specific tissue microstructure or rigid atlas demarcations may further differentiate already varied inter-atlas subnuclei.

### 4.3 Clinical validation

The correspondence of the atlases and segmentation approaches with patient outcomes was analyzed to evaluate their clinical applicability. Comparing motor and sensory activation to known therapeutic and side-effect outcomes provided a clinically relevant benchmark that was preferable to comparing the resulting segmentations to the atlases that originally informed them.

The border between motor and sensory subnuclei is theorized to affect the prevalence of sustained paresthesia resulting from VIM DBS (Keane et al., 2012, Kuncel et al., 2008). High VTA-motor overlap and low VTA-sensory overlap were expected for therapeutic stimulation (Malaga et al., 2022, Middlebrooks et al., 2018), and ABS showed this in all atlases, whereas DTIBS did so in only half. VTA-sensory overlap was expected to be greater for paresthesia VTAs compared to tremor reduction VTAs (Halász et al., 2024), and this was observed in more atlases when thalamic segmentation was performed with ABS rather than DTIBS. Additionally, DTIBS with the Ding and Saranathan atlases showed significantly greater VTA-sensory overlap for therapeutic VTAs compared to paresthesia VTAs, the opposite of what was expected. The wider interquartile range for VTA-sensory overlap across all atlases using DTIBS compared to ABS also revealed the higher variability of DTIBS, as the DTI data utilized was patient-specific. The generalizability of ABS indicated that the choice of atlas was more important for DTIBS, as the number and location of the seed points for the clustering algorithm was atlas-dependent.

ABS produced a significantly larger motor subnuclei volume in four atlases and a smaller sensory subnuclei volume in five atlases compared to DTIBS. The larger motor volume associated with ABS likely resulted in increased VTA-motor overlap, thereby explaining the greater motor activation for therapeutic outcomes observed. DTIBS with the Iglesias atlas produced the smallest sensory volume, which likely explains the minimal sensory activation observed for that particular atlas.

The atlases that performed well for therapeutic outcomes using both segmentation techniques (Iglesias, Jakab, and Saranathan) differed in their number of motor, sensory, and total subnuclei. Those that performed well for paresthesia outcomes (Ewert, Ilinsky, and Jakab) also differed in their number of subnuclei. The Jakab atlas was the only atlas that showed the expected VTA correspondence for both clinical outcomes using either ABS or DTIBS, making it the most clinically applicable atlas of the six analyzed. The Morel atlas (Morel, 2007), which the Jakab atlas is based on, was coincidentally selected as the singular atlas used in a previous study by our group that found DTIBS was more effective than ABS at explaining paresthesia outcomes and as effective at explaining therapeutic outcomes (Malaga et al., 2022). The present study revealed this finding cannot be generalized to any atlas, highlighting that the effectiveness of both segmentation approaches depends on the chosen atlas and suggesting that a complex interplay between atlas and segmentation choice exists.

Given their integration with the Lead-DBS toolbox, all of these atlases are applicable to DBS cohorts. Some atlases were constructed with DBS applications in mind, such as surgical planning and clinical programming (Ewert et al., 2018, Iglesias et al., 2018, Ilinsky et al., 2018, Su et al., 2019). The Morel atlas has been cited in numerous DBS analysis studies; so has the Ding atlas, albeit to a lesser extent. While applicability to DBS is shared by all atlases, usage and validation in DBS studies differs. Our study addresses this by investigating atlas correspondence with clinical outcomes.

The optimal target for DBS in ET likely is not restricted to motor thalamus. Using connectivity-based techniques, the dentato-rubro-thalamic tract (DRTT) has been identified as a potentially more effective stimulation target than the thalamic VIM (Akram et al., 2018, Middlebrooks et al., 2021). Notably, many previously reported “sweet spots” overlap with the DRTT to some extent (Middlebrooks et al., 2021). This includes the posterior subthalamic area, specifically near the inferior borders of the VIM and sensory thalamus (Al-Fatly et al., 2019). This network-level approach to targeting uses connectome data, which can be normative (like an atlas) (Al-Fatly et al., 2019, Middlebrooks et al., 2021) or individualized (Akram et al., 2018, Middlebrooks et al., 2018), to identify fiber connections (rather than individual nuclei) associated with clinical response. Recently, it has been shown that network connectivity can vary significantly depending on the connectome used, with little agreement across connectomes (Mehta et al., 2025). This finding is especially relevant to the present study, which demonstrated that thalamic segmentation depends on the atlas used and that clinical correspondence varied across atlases. As DBS modeling becomes more widely utilized due to improved accessibility through platforms such as Lead-DBS, it is increasingly important for researchers to recognize how atlas choice (whether it be the thalamus or the entire brain) can influence model predictions and the interpretation of results.

### 4.4 Limitations and future work

While DTIBS generally showed inferior performance compared to ABS, this was not uniformly the case across all atlases, suggesting that DTIBS performance may be partially dependent on atlas-specific factors. Several factors (not all atlas-specific) likely contributed to the variable performance of DTIBS. First, anatomical variability across atlases can affect the DTIBS results. Because DTIBS relies on the number and location of seed points based on atlas-defined nuclei for clustering, inconsistencies or inaccuracies in the atlas delineations can reduce segmentation accuracy. Some atlases may align better with true individual anatomy than others, resulting in improved DTIBS performance. The *k*-means clustering algorithm in this study and others (Battistella et al., 2017, Wiegell et al., 2003) weighted physical distance and DTI-derived tissue properties equally. This was done to enable fair comparison. However, refining the weights of these features for each atlas could improve individual performance using DTIBS. Interhemispheric anatomical differences within atlases may also impact DTIBS results, although likely to a lesser degree than atlas-to-atlas variability. This was accounted for by matching the hemisphere of the atlas thalamus to that of the patient. Nonetheless, refining DTIBS parameters on a per-hemisphere basis could further improve performance.

Second, the quality of the clinical DTI plays a critical role. DTIBS depends on a multistep image registration pipeline before segmentation occurs (Malaga et al., 2022), and registration errors can propagate through this process. Higher-resolution imaging may mitigate this issue by providing more detailed and distinct anatomical landmarks. Furthermore, previous work has shown that DTI obtained at a greater magnetic field strength can distinguish subnuclei (Oishi et al., 2025). Incorporating higher-resolution DTI into DTIBS may enhance its thalamic segmentation capabilities. However, acquiring such data often requires longer scan times or higher-field-strength scanners, which are not always clinically feasible.

Third, the DTIBS clustering algorithm segments patient thalami based on the geometric proximity of voxels and the similarity of the tensors associated with them (Malaga et al., 2022). This approach assigns each voxel to a cluster independently and therefore may not capture the continuous, anatomically nuanced boundaries between nuclei as effectively as ABS, which applies predefined anatomical segmentations. Other thalamic segmentation techniques utilizing spectral clustering, neural networks, and tractography have demonstrated success based on atlas similarity (Ji et al., 2016, Johansen-Berg et al., 2005, Majdi et al., 2020). However, the *k*-means clustering algorithm from our previous work (Malaga et al., 2022) was chosen for this study to enable direct comparison and better isolate the effect of atlas choice. Incorporating geometric features from atlas subnuclei alongside DTI-derived features (Glaister et al., 2017) could provide additional guidance for DTIBS, thereby improving its segmentation results.

Last, the relatively small sample size, single-center dataset, and analysis of broad functional regions may have limited the statistical power to detect subtle effects and reduced the generalizability of the findings to broader or more heterogeneous ET patient populations. The treatment of clinical outcome as a categorical variable might also overestimate the degree of improvement, so more objective evidence would strengthen the results. Nonetheless, this study highlights the importance of considering different atlases and segmentation approaches when evaluating predictions from DBS analyses (Mehta et al., 2025). Further investigation with larger, more diverse cohorts, individual subnuclei, and quantitative measures of tremor reduction (Akram et al., 2018, Al-Fatly et al., 2019, Middlebrooks et al., 2021) and sustained paresthesia is needed to fully compare and validate the clinical applicability of DTIBS and ABS used alongside different atlases.

## 5 Conclusions

An extensive cross-atlas analysis was performed among six prominent thalamic atlases and two thalamic segmentation approaches, revealing that traditional ABS aligned more closely with clinical outcomes than data-driven DTIBS across atlases. DTIBS has the potential to develop more personalized treatment strategies for ET patients undergoing VIM DBS, but this segmentation technique needs further refinement for it to definitively surpass ABS. The Jakab atlas properly corresponded with the therapeutic and side-effect outcomes using both segmentation approaches, resulting in it being the most clinically applicable atlas analyzed in this study.

## Funding sources

This work was supported by the Costa Healthcare Research & Design Fund (Karlo A. Malaga and Mathew E. Zilberman) and the Swanson Fellowship in the Sciences and Engineering (Karlo A. Malaga) at Bucknell University.

## CRediT authorship contribution statement

**Mathew E. Zilberman:** Writing – original draft, Visualization, Validation, Software, Methodology, Investigation, Funding acquisition, Formal analysis, Data curation, Conceptualization. **Kelvin L. Chou:** Writing – review & editing, Resources. **Parag G. Patil:** Writing – review & editing, Resources. **Karlo A. Malaga:** Writing – review & editing, Validation, Supervision, Resources, Project administration, Methodology, Investigation, Funding acquisition, Formal analysis, Data curation, Conceptualization.

## Declaration of competing interests

Kelvin L. Chou reports personal fees from Abbott for participating as a faculty member in education sessions about DBS. Parag G. Patil serves as a consultant to NeuroOne Medical Technologies Corporation and Epiminder. Mathew E. Zilberman and Karlo A. Malaga declare no competing interests related to this work.

## Data Availability

Data will be made available on request.
