# Supplementary material for: Comparison of thalamic atlases and segmentation techniques in defining motor and sensory nuclei for deep brain stimulation targeting in essential tremor
Source: Neuroimage Clin. 2025 Sep 27;48:103887. doi: 10.1016/j.nicl.2025.103887 (PMC12512978; doi:10.1016/j.nicl.2025.103887)
Supplement: Supplementary Data 1 [file mmc1.docx]

**Supplementary Material**

Supplementary Table 1. Programming logs containing stimulation settings eliciting tremor suppression and paresthesia for all patients (n = 22).

| **Pat** | **Side** | **Final Settings** | | | | | **Contact** | **Tremor Suppression** | | | | | | | | | **Paresthesia** | | | | | | |
| --- | --- | --- | --- | --- | --- | --- | --- | --- | --- | --- | --- | --- | --- | --- | --- | --- | --- | --- | --- | --- | --- | --- | --- |
|  |  | **Contact** | **Amp** | | **PW** | **Freq** |  | **Better** | | | **Minimal** | | | **Absent** | | | **Transient** | | | | **Sustained** | | |
|  |  |  | **mA** | **V** | **μs** | **Hz** |  | **mA** | **V** | **Part** | **mA** | **V** | **Part** | **mA** | **V** | **Part** | **mA** | **V** | **Part** | **mA** | | **V** | **Part** |
| 1 | L |  |  |  |  |  | 0 |  |  |  |  |  |  |  |  |  | 1 |  | RA | 1.5 | |  | RA |
|  |  |  |  |  |  |  | 1 | 1 |  | R. index finger | 1.5 |  | R. index finger |  |  |  | 1 |  | RA | 2 | |  | RA |
|  |  |  |  |  |  |  | 2 | 1 |  | R. index finger |  |  |  | 2.5 |  | R. index finger |  |  |  |  | |  |  |
|  |  | 2A- | 1.5 |  | 60 | 130 | 2A | 1 |  | R. index finger | 1.5 |  | R. index finger | 2 |  | R. index finger | 1.5 |  | RA |  | |  |  |
|  |  |  |  |  |  |  | 2A |  |  |  |  |  |  |  |  |  | 2 |  | RA, face | 2.5 | |  | RA |
|  |  |  |  |  |  |  | 3 | 3.5 |  | R. index finger |  |  |  |  |  |  | 5 |  | RA, face |  | |  |  |
| 2 | R |  |  |  |  |  |  |  |  |  |  |  |  |  |  |  |  |  |  |  | |  |  |
|  |  |  |  |  |  |  | 0 |  | 1 |  |  | 1.5 |  |  |  |  |  | 1 | LA, |  | | 4 | LA, LL |
|  |  |  |  |  |  |  | 0 |  |  |  |  |  |  |  |  |  |  | 1.5-3.5 | LA, LL |  | |  |  |
|  |  |  |  |  |  |  | 1 |  | 1.5 |  |  | 2.5 |  |  |  |  |  | 2.5 | LA |  | |  |  |
|  |  |  |  |  |  |  | 1 |  |  |  |  |  |  |  |  |  |  | 3-3.5 | LA, LL |  | |  |  |
|  |  | 2- |  | 2.5 | 60 | 130 | 2 |  | 1.5 |  |  | 2.5 |  |  |  |  |  | 5 | LA, LL |  | |  |  |
|  |  |  |  |  |  |  | 3 |  | 3.5 |  |  |  |  |  |  |  |  |  |  |  | |  |  |
| 3 | L |  |  |  |  |  |  |  |  |  |  |  |  |  |  |  |  |  |  |  | |  |  |
|  |  |  |  |  |  |  | 0 |  |  |  | 1 |  | UE |  |  |  | 0.5 |  | RA | 1-1.5 | |  | RA, face |
|  |  |  |  |  |  |  | 1 |  |  |  | 1 |  | UE | 2 |  | UE | 1 |  | RA |  | |  |  |
|  |  |  |  |  |  |  | 1 |  |  |  |  |  |  |  |  |  | 2 |  | RL | 2.5 | |  | RA, RL |
|  |  |  |  |  |  |  | 2 | 1 |  | UE | 1.5 |  | UE | 2.5 |  | UE | 2.5 |  | RA, RL |  | |  |  |
|  |  | 3ABC- | 2 |  | 60 | 130 | 3 | 3.5 |  | UE | 5 |  | UE |  |  |  |  |  |  |  | |  |  |
| 4 | L |  |  |  |  |  |  |  |  |  |  |  |  |  |  |  |  |  |  |  | |  |  |
|  |  |  |  |  |  |  | 0 |  |  |  |  |  |  | 1.5 |  |  | 1-1.5 |  | RA | 2 | |  | RA, face |
|  |  |  |  |  |  |  | 1 | 1.5 |  |  | 2.5 |  |  |  |  |  | 2-2.5 |  | RA | 3 | |  | RA, face |
|  |  |  |  |  |  |  | 2 |  |  |  |  |  |  |  |  |  |  |  |  | 3.5 | |  | RA |
|  |  |  |  |  |  |  | 2 | 1.5 |  |  | 2.5 |  |  |  |  |  |  |  |  | 4 | |  | face |
|  |  | 3ABC- | 2.5 |  | 60 | 130 | 3 |  |  |  |  |  |  | 3 |  |  | 4.5 |  | RA |  | |  |  |
| 5 | L |  |  |  |  |  |  |  |  |  |  |  |  |  |  |  |  |  |  |  | |  |  |
|  |  | 0- | 1.5 |  | 60 | 130 | 0 | 0.5 |  |  |  |  |  | 2 |  |  | 1.5 |  | RA |  | |  |  |
|  |  |  |  |  |  |  | 1 | 1.5 |  |  |  |  |  | 2 |  |  | 2 |  | RA |  | |  |  |
|  |  |  |  |  |  |  | 2 | 2.5 |  |  | 3 |  |  | 3.5 |  |  |  |  |  |  | |  |  |
|  |  |  |  |  |  |  | 3 | 0.5 |  |  | 4 |  |  |  |  |  |  |  |  |  | |  |  |
| 6 | L |  |  |  |  |  |  |  |  |  |  |  |  |  |  |  |  |  |  |  | |  |  |
|  |  |  |  |  |  |  | 0 |  | 1.5 |  |  | 2 |  |  |  |  |  | 1-2 | RA |  | | 2.5 | RA |
|  |  |  |  |  |  |  | 1 |  | 1 |  |  | 1.5 |  |  |  |  |  | 2.5-4.5 | RA |  | |  |  |
|  |  |  |  |  |  |  | 2 |  | 1.5 |  |  | 2 |  |  |  |  |  |  |  |  | |  |  |
|  |  | 3- |  | 1.5 | 60 | 130 | 3 |  | 1 |  |  |  |  |  |  |  |  |  |  |  | |  |  |
| 7 | L |  |  |  |  |  |  |  |  |  |  |  |  |  |  |  |  |  |  |  | |  |  |
|  |  |  |  |  |  |  | 0 |  | 1 |  |  | 1.5 |  |  |  |  |  | 1-2 | RA, RL |  | | 2.5 | RA, RL |
|  |  |  |  |  |  |  | 1 |  |  |  |  | 1 |  |  |  |  |  | 1 |  |  | | 2 | RA, RL |
|  |  |  |  |  |  |  | 1 |  |  |  |  |  |  |  |  |  |  | 1.5 | RA, foot, face |  | |  |  |
|  |  | 2- |  | 1.5 | 60 | 130 | 2 |  |  |  |  | 1 |  |  | 3 |  |  | 1-1.5 | RA |  | | 4.5 |  |
|  |  |  |  |  |  |  | 2 |  |  |  |  |  |  |  |  |  |  | 2 | RA, face |  | |  |  |
|  |  |  |  |  |  |  | 3 |  | 1 |  |  | 3 |  |  |  |  |  | 4 | RA |  | |  |  |
| 8 | R |  |  |  |  |  |  |  |  |  |  |  |  |  |  |  |  |  |  |  | |  |  |
|  |  |  |  |  |  |  | 0 |  |  |  |  | 1.5 |  |  |  |  |  | 1 | LA |  | | 2 | LA |
|  |  |  |  |  |  |  | 1 |  |  |  |  | 1 |  |  | 1.5 |  |  | 1.5 | LA |  | | 2 | LA, mouth |
|  |  | 2- |  | 2 | 60 | 130 | 2 |  | 1 |  |  | 2 |  |  | 2.5 |  |  | 2.5 | LA |  | | 3.5 | LA, mouth |
|  |  |  |  |  |  |  | 3 |  | 2.5 |  |  |  |  |  | 3.5 |  |  | 4.5 | L. mouth |  | | 5 |  |
| 9 | L |  |  |  |  |  |  |  |  |  |  |  |  |  |  |  |  |  |  |  | |  |  |
|  |  |  |  |  |  |  | 0 |  | 1.5 | RA |  |  |  |  |  |  |  | 1 | RA |  | |  |  |
|  |  |  |  |  |  |  | 0 |  |  |  |  |  |  |  |  |  |  | 1.5 | RA, R. foot |  | | 2 | RA |
|  |  |  |  |  |  |  | 1 |  |  |  |  |  |  |  | 1.5 | RA |  |  |  |  | | 3.5 | RA, RL |
|  |  | 2- |  | 1.5 | 60 | 130 | 2 |  |  |  |  |  |  |  | 2 | RA |  | 3 | RA |  | | 3.5 | RA |
|  |  |  |  |  |  |  | 3 |  |  |  |  |  |  |  | 2-5 | RA |  |  |  |  | |  |  |
| 10 | L |  |  |  |  |  |  |  |  |  |  |  |  |  |  |  |  |  |  |  | |  |  |
|  |  |  |  |  |  |  | 0 |  |  |  |  |  |  |  |  |  |  | 1-2 | RA |  | | 2.5 | RA, RL |
|  |  |  |  |  |  |  | 1 |  |  |  |  |  |  |  |  |  |  | 1.5 | RA |  | |  |  |
|  |  |  |  |  |  |  | 1 |  |  |  |  |  |  |  |  |  |  | 2.5-3 | RA, RL |  | |  |  |
|  |  | 2- |  | 2 | 60 | 130 | 2 |  | 2 |  |  |  |  |  |  |  |  | 2.5 | RA |  | |  |  |
|  |  |  |  |  |  |  | 2 |  |  |  |  |  |  |  |  |  |  | 4 | RA, RL, R. face |  | |  |  |
|  |  |  |  |  |  |  | 3 |  |  |  |  |  |  |  |  |  |  |  |  |  | |  |  |
| 11 | L |  |  |  |  |  |  |  |  |  |  |  |  |  |  |  |  |  |  |  | |  |  |
|  |  |  |  |  |  |  | 0 |  |  |  |  |  |  |  | 1 | RA |  |  |  |  | | 2.5 | RA |
|  |  |  |  |  |  |  | 1 |  |  |  |  | 1.5 | RA |  | 2 | RA |  |  |  |  | |  |  |
|  |  | 2- |  | 2 | 60 | 130 | 2 |  |  |  |  | 1.5 | RA |  | 2 | RA |  |  |  |  | |  |  |
|  |  |  |  |  |  |  | 3 |  | 4 | RA |  |  |  |  |  |  |  |  |  |  | |  |  |
| 12 | L |  |  |  |  |  |  |  |  |  |  |  |  |  |  |  |  |  |  |  | |  |  |
|  |  |  |  |  |  |  | 0 |  | 1 |  |  |  |  |  | 1.5 |  |  | 1-3 | RA |  | |  |  |
|  |  |  |  |  |  |  | 1 |  | 1-4.5 |  |  |  |  |  |  |  |  | 3-5 | RA |  | |  |  |
|  |  | 2- |  | 2.5 | 60 | 130 | 2 |  | 1 |  |  |  |  |  | 2 |  |  | 5 |  |  | |  |  |
|  |  |  |  |  |  |  | 3 |  | 2 |  |  |  |  |  | 3.5 |  |  |  |  |  | |  |  |
| 13 | R |  |  |  |  |  |  |  |  |  |  |  |  |  |  |  |  |  |  |  | |  |  |
|  |  |  |  |  |  |  | 0 |  | 1 |  |  |  |  |  | 2 |  |  | 1-2.5 | LA |  | |  |  |
|  |  |  |  |  |  |  | 0 |  |  |  |  |  |  |  |  |  |  | 3 | LA, RA |  | |  |  |
|  |  | 1- |  | 2 | 60 | 130 | 1 |  |  |  |  | 1 |  |  | 2 |  |  | 3-3.5 | LA |  | |  |  |
|  |  |  |  |  |  |  | 2 |  | 1.5 |  |  |  |  |  | 5 |  |  |  |  |  | |  |  |
|  |  |  |  |  |  |  | 3 |  | 1 |  |  | 5 |  |  |  |  |  |  |  |  | |  |  |
| 14 | L |  |  |  |  |  |  |  |  |  |  |  |  |  |  |  |  |  |  |  | |  |  |
|  |  |  |  |  |  |  | 0 |  |  |  |  |  |  |  |  |  |  | 0.5-1 | RA |  | |  |  |
|  |  | 1- |  | 1.5 | 60 | 130 | 1 |  |  |  |  |  |  |  | 1.5 | RA |  | 1.5-2.5 | RA |  | |  |  |
|  |  |  |  |  |  |  | 2 |  |  |  |  |  |  |  | 1.5 | RA |  |  |  |  | |  |  |
|  |  |  |  |  |  |  | 3 |  |  |  |  |  |  |  | 2.5 | RA |  | 2.5-3 | RA |  | |  |  |
| 15 | L |  |  |  |  |  |  |  |  |  |  |  |  |  |  |  |  |  |  |  | |  |  |
|  |  | 0- |  | 2 | 60 | 130 | 0 |  |  |  |  | 1.5 | RA |  |  |  |  | 1-4.5 | RA |  | |  |  |
|  |  |  |  |  |  |  | 1 |  |  |  |  | 1.5 | RA |  |  |  |  | 1-4.5 | RA |  | |  |  |
|  |  |  |  |  |  |  | 2 |  |  |  |  | 3-5 | RA |  |  |  |  |  |  |  | |  |  |
|  |  |  |  |  |  |  | 3 |  |  |  |  |  |  |  |  |  |  |  |  |  | |  |  |
| 16 | L |  |  |  |  |  |  |  |  |  |  |  |  |  |  |  |  |  |  |  | |  |  |
|  |  |  |  |  |  |  | 0 |  | 1 |  |  |  |  |  | 1.5 |  |  | 2-3 | RA |  | |  |  |
|  |  | 1- |  | 1.5 | 60 | 130 | 1 |  |  |  |  |  |  |  | 1.5 |  |  | 2.5-3 | RA |  | |  |  |
|  |  |  |  |  |  |  | 2 |  |  |  |  |  |  |  | 2 |  |  | 2.5-4.5 | RA |  | |  |  |
|  |  |  |  |  |  |  | 3 |  |  |  |  |  |  |  |  |  |  |  |  |  | |  |  |
| 17 | L |  |  |  |  |  |  |  |  |  |  |  |  |  |  |  |  |  |  |  | |  |  |
|  |  | 0- |  | 1 | 60 | 130 | 0 |  |  |  |  |  |  |  | 1 | RA |  | 0.5-2 | RA, tongue |  | |  |  |
|  |  |  |  |  |  |  | 1 |  | 1 |  |  |  |  |  | 1.5 | RA |  | 1-2 | RA |  | |  |  |
|  |  |  |  |  |  |  | 2 |  |  |  |  |  |  |  | 1.5 | RA |  | 2 | RA |  | |  |  |
|  |  |  |  |  |  |  | 3 |  |  |  |  |  |  |  | 3.5 |  |  | 4 | RA |  | |  |  |
| 18 | L |  |  |  |  |  |  |  |  |  |  |  |  |  |  |  |  |  |  |  | |  |  |
|  |  |  |  |  |  |  | 0 |  |  |  |  |  |  |  |  |  |  |  |  |  | | 1 | RA |
|  |  |  |  |  |  |  | 1 |  | 1 | RA |  |  |  |  |  |  |  | 1 | RA, R. face |  | | 1.5 | RA |
|  |  |  |  |  |  |  | 2 |  |  |  |  |  |  |  | 1.5 | RA |  |  |  |  | | 2 | RA, face |
|  |  | 3- |  | 1.5 | 60 | 130 | 3 |  |  |  |  | 1.5 | RA |  |  |  |  |  |  |  | |  |  |
| 19 | L |  |  |  |  |  |  |  |  |  |  |  |  |  |  |  |  |  |  |  | |  |  |
|  |  |  |  |  |  |  | 0 |  |  |  |  |  |  |  |  |  |  |  |  |  | |  |  |
|  |  |  |  |  |  |  | 1 |  |  |  |  |  |  |  |  |  |  |  |  |  | |  |  |
|  |  |  |  |  |  |  | 2 |  |  |  |  |  |  |  |  |  |  |  |  |  | |  |  |
|  |  | 3- |  | 1 | 60 | 130 | 3 |  | 1 |  |  |  |  |  |  |  |  |  |  |  | |  |  |
| 20 | L |  |  |  |  |  |  |  |  |  |  |  |  |  |  |  |  |  |  |  | |  |  |
|  |  |  |  |  |  |  | 0 |  |  |  |  |  |  |  |  |  |  |  |  |  | |  |  |
|  |  |  |  |  |  |  | 1 |  |  |  |  |  |  |  |  |  |  |  |  |  | |  |  |
|  |  | 2- |  | 2 | 60 | 130 | 2 |  | 2 |  |  |  |  |  |  |  |  |  |  |  | |  |  |
|  |  |  |  |  |  |  | 3 |  |  |  |  |  |  |  |  |  |  |  |  |  | |  |  |
| 21 | L |  |  |  |  |  |  |  |  |  |  |  |  |  |  |  |  |  |  |  | |  |  |
|  |  | 0- |  | 2 | 60 | 130 | 0 |  | 2 |  |  |  |  |  |  |  |  |  |  |  | |  |  |
|  |  |  |  |  |  |  | 1 |  |  |  |  |  |  |  |  |  |  |  |  |  | |  |  |
|  |  |  |  |  |  |  | 2 |  |  |  |  |  |  |  |  |  |  |  |  |  | |  |  |
|  |  |  |  |  |  |  | 3 |  |  |  |  |  |  |  |  |  |  |  |  |  | |  |  |
| 22 | L |  |  |  |  |  |  |  |  |  |  |  |  |  |  |  |  |  |  |  | |  |  |
|  |  |  |  |  |  |  | 0 |  |  |  |  |  |  |  |  |  |  |  |  |  | |  |  |
|  |  |  |  |  |  |  | 1 |  |  |  |  |  |  |  |  |  |  |  |  |  | |  |  |
|  |  |  |  |  |  |  | 2 |  |  |  |  |  |  |  |  |  |  |  |  |  | |  |  |
|  |  | 3- |  | 1 | 60 | 130 | 3 |  | 1 |  |  |  |  |  |  |  |  |  |  |  | |  |  |

Abbreviations: Pat, patient; Amp, amplitude; PW, pulse width; Freq, frequency; R, right; L, left; RA, right arm; LA, left arm; RL, right leg; LL, left leg; UE, upper extremity.

Supplementary Table 2. Atlas files with thalamic subnuclei labeled*.

| **Index** | **Saranathan 2019** | **Iglesias 2018** | **Jakab 2008** | **Ilinsky 2017** | **Ewert 2017** | **Ding 2020** |
| --- | --- | --- | --- | --- | --- | --- |
| 1 | AV.nii.gz' | AV.nii.gz' | AD.nii.gz' | lf.nii.gz' | STN.nii.gz' | 3V.nii.gz' |
| 2 | CM.nii.gz' | CL.nii.gz' | AM.nii.gz' | SN.nii.gz' | STN_motor.nii.gz' | 4V.nii.gz' |
| 3 | Hb.nii.gz' | CM.nii.gz' | AV.nii.gz' | MD.nii.gz' | STN_associative.nii.gz' | AAA.nii.gz' |
| 4 | LGN.nii.gz' | CeM.nii.gz' | CL.nii.gz' | MI.nii.gz' | STN_limbic.nii.gz' | AG.nii.gz' |
| 5 | MD-Pf.nii.gz' | L-Sg.nii.gz' | CM.nii.gz' | eml.nii.gz' | GPi.nii.gz' | AHA.nii.gz' |
| 6 | MGN.nii.gz' | LD.nii.gz' | CeM.nii.gz' | Hbm.nii.gz' | GPi_sensorimotor.nii.gz' | AMY.nii.gz' |
| 7 | MTT.nii.gz' | LGN.nii.gz' | Hb.nii.gz' | A.nii.gz' | GPi_primarymotor.nii.gz' | ANC.nii.gz' |
| 8 | Pul.nii.gz' | LP.nii.gz' | LD.nii.gz' | BasalForebrain.nii.gz' | GPi_premotor.nii.gz' | AON.nii.gz' |
| 9 | THALAMUS.nii.gz' | MDl.nii.gz' | LGNmc.nii.gz' | CdPutVSt.nii.gz' | GPi_sensory.nii.gz' | AOrG.nii.gz' |
| 10 | VA.nii.gz' | MDm.nii.gz' | LGNpc.nii.gz' | MGC.nii.gz' | GPi_postparietal.nii.gz' | APH.nii.gz' |
| 11 | VLP.nii.gz' | MGN.nii.gz' | LP.nii.gz' | MDCL.nii.gz' | GPi_occipital.nii.gz' | AnG.nii.gz' |
| 12 | VLPd.nii.gz' | MV(Re).nii.gz' | Li.nii.gz' | PPN.nii.gz' | GPi_temporal.nii.gz' | Aq.nii.gz' |
| 13 | VLPv.nii.gz' | Pc.nii.gz' | MDmc.nii.gz' | CMPf.nii.gz' | GPi_prefrontal.nii.gz' | BF.nii.gz' |
| 14 | VLa.nii.gz' | Pf.nii.gz' | MDpc.nii.gz' | GPm.nii.gz' | RN.nii.gz' | BL.nii.gz' |
| 15 | VPL.nii.gz' | Pt.nii.gz' | MGN.nii.gz' | GPl.nii.gz' | GPe.nii.gz' | BM.nii.gz' |
| 16 |  | PuA.nii.gz' | MV.nii.gz' | Hypothalamus.nii.gz' | 1.nii.gz' | BNST.nii.gz' |
| 17 |  | PuI.nii.gz' | Pf.nii.gz' | Hbl.nii.gz' | 2.nii.gz' | CBL.nii.gz' |
| 18 |  | PuL.nii.gz' | Po.nii.gz' | ot.nii.gz' | 3.nii.gz' | CBPV.nii.gz' |
| 19 |  | PuM.nii.gz' | PuA.nii.gz' | ic.nii.gz' | 4.nii.gz' | CBV.nii.gz' |
| 20 |  | VA.nii.gz' | PuI.nii.gz' | LG.nii.gz' | 6.nii.gz' | CEN.nii.gz' |
| 21 |  | VAmc.nii.gz' | PuL.nii.gz' | LD.nii.gz' | 7.nii.gz' | CM.nii.gz' |
| 22 |  | VLa.nii.gz' | PuM.nii.gz' | Li.nii.gz' | 8.nii.gz' | CaB.nii.gz' |
| 23 |  | VLp.nii.gz' | Pv.nii.gz' | Midbrain.nii.gz' | 9.nii.gz' | CaH.nii.gz' |
| 24 |  | VM.nii.gz' | RN.nii.gz' | MG.nii.gz' | 10.nii.gz' | CaT.nii.gz' |
| 25 |  | VPL.nii.gz' | SG.nii.gz' | PC.nii.gz' | 13.nii.gz' | CbDN.nii.gz' |
| 26 |  |  | STh.nii.gz' | Po.nii.gz' | 14.nii.gz' | CgGc.nii.gz' |
| 27 |  |  | VAmc.nii.gz' | Pul.nii.gz' | 16.nii.gz' | CgGr.nii.gz' |
| 28 |  |  | VApc.nii.gz' | Rt.nii.gz' | 17.nii.gz' | Cla.nii.gz' |
| 29 |  |  | VLa.nii.gz' | alfrfxmtpc.nii.gz' | 19.nii.gz' | CoA.nii.gz' |
| 30 |  |  | VLpd.nii.gz' | MB.nii.gz' | 20.nii.gz' | CoP.nii.gz' |
| 31 |  |  | VLpv.nii.gz' | VPi.nii.gz' | 21.nii.gz' | Cun.nii.gz' |
| 32 |  |  | VM.nii.gz' | Sch.nii.gz' | 22.nii.gz' | DLG.nii.gz' |
| 33 |  |  | VPI.nii.gz' | R.nii.gz' | 23.nii.gz' | FI.nii.gz' |
| 34 |  |  | VPLa.nii.gz' | STN.nii.gz' | 24.nii.gz' | FMG.nii.gz' |
| 35 |  |  | VPLp.nii.gz' | VAn.nii.gz' | 25.nii.gz' | FP.nii.gz' |
| 36 |  |  | VPM.nii.gz' | VAp.nii.gz' | 26.nii.gz' | FWM.nii.gz' |
| 37 |  |  | mtt.nii.gz' | VLdVLv.nii.gz' | 27.nii.gz' | FrO.nii.gz' |
| 38 |  |  | sPf.nii.gz' | M.nii.gz' | 28.nii.gz' | FuGo.nii.gz' |
| 39 |  |  |  | VPlVPm.nii.gz' | 29.nii.gz' | FuGt.nii.gz' |
| 40 |  |  |  | MIa.nii.gz' | 35.nii.gz' | GPe.nii.gz' |
| 41 |  |  |  | Connectivetissue.nii.gz' | 36.nii.gz' | GPi.nii.gz' |
| 42 |  |  |  | acbcbsc.nii.gz' | 37.nii.gz' | HN.nii.gz' |
| 43 |  |  |  |  | 40.nii.gz' | HTH.nii.gz' |
| 44 |  |  |  |  | 41.nii.gz' | HTHma.nii.gz' |
| 45 |  |  |  |  | 47.nii.gz' | HTHpo.nii.gz' |
| 46 |  |  |  |  | 49.nii.gz' | HTHso.nii.gz' |
| 47 |  |  |  |  | 51.nii.gz' | HTHtub.nii.gz' |
| 48 |  |  |  |  | 52.nii.gz' | HWM.nii.gz' |
| 49 |  |  |  |  | 53.nii.gz' | HiB.nii.gz' |
| 50 |  |  |  |  | 60.nii.gz' | HiH.nii.gz' |
| 51 |  |  |  |  | 61.nii.gz' | HiT.nii.gz' |
| 52 |  |  |  |  | 63.nii.gz' | IC.nii.gz' |
| 53 |  |  |  |  | 64.nii.gz' | IFGop.nii.gz' |
| 54 |  |  |  |  | 66.nii.gz' | IFGtr.nii.gz' |
| 55 |  |  |  |  | 67.nii.gz' | IO.nii.gz' |
| 56 |  |  |  |  | 68.nii.gz' | IOG.nii.gz' |
| 57 |  |  |  |  | 70.nii.gz' | ITG.nii.gz' |
| 58 |  |  |  |  | 71.nii.gz' | IsCPH.nii.gz' |
| 59 |  |  |  |  | 73.nii.gz' | LD.nii.gz' |
| 60 |  |  |  |  | 74.nii.gz' | LI.nii.gz' |
| 61 |  |  |  |  | 75.nii.gz' | LIG.nii.gz' |
| 62 |  |  |  |  | 76.nii.gz' | LOG.nii.gz' |
| 63 |  |  |  |  | 81.nii.gz' | LOrG.nii.gz' |
| 64 |  |  |  |  | 86.nii.gz' | LP.nii.gz' |
| 65 |  |  |  |  | 87.nii.gz' | La.nii.gz' |
| 66 |  |  |  |  | 88.nii.gz' | LingG.nii.gz' |
| 67 |  |  |  |  | 89.nii.gz' | MD.nii.gz' |
| 68 |  |  |  |  | 90.nii.gz' | MFG.nii.gz' |
| 69 |  |  |  |  | 91.nii.gz' | MG.nii.gz' |
| 70 |  |  |  |  | 92.nii.gz' | MOrG.nii.gz' |
| 71 |  |  |  |  | 93.nii.gz' | MTG.nii.gz' |
| 72 |  |  |  |  | 94.nii.gz' | MTgm.nii.gz' |
| 73 |  |  |  |  | 95.nii.gz' | Me.nii.gz' |
| 74 |  |  |  |  | 96.nii.gz' | MiN.nii.gz' |
| 75 |  |  |  |  | 97.nii.gz' | MoPy.nii.gz' |
| 76 |  |  |  |  | 98.nii.gz' | MoTg.nii.gz' |
| 77 |  |  |  |  | 99.nii.gz' | NAC.nii.gz' |
| 78 |  |  |  |  | 100.nii.gz' | OB.nii.gz' |
| 79 |  |  |  |  | 101.nii.gz' | OP.nii.gz' |
| 80 |  |  |  |  | 102.nii.gz' | PCLc.nii.gz' |
| 81 |  |  |  |  | 103.nii.gz' | PCLr.nii.gz' |
| 82 |  |  |  |  | 104.nii.gz' | PLP.nii.gz' |
| 83 |  |  |  |  | 105.nii.gz' | PLT.nii.gz' |
| 84 |  |  |  |  | 106.nii.gz' | POrG.nii.gz' |
| 85 |  |  |  |  | 107.nii.gz' | PPH.nii.gz' |
| 86 |  |  |  |  | 108.nii.gz' | PRG.nii.gz' |
| 87 |  |  |  |  | 109.nii.gz' | PTR.nii.gz' |
| 88 |  |  |  |  | 110.nii.gz' | PaCG.nii.gz' |
| 89 |  |  |  |  | 111.nii.gz' | PaO.nii.gz' |
| 90 |  |  |  |  | 112.nii.gz' | Pf.nii.gz' |
| 91 |  |  |  |  | 113.nii.gz' | Pin.nii.gz' |
| 92 |  |  |  |  | 114.nii.gz' | Pir.nii.gz' |
| 93 |  |  |  |  | 115.nii.gz' | PnBa.nii.gz' |
| 94 |  |  |  |  | 116.nii.gz' | PnTg.nii.gz' |
| 95 |  |  |  |  | 117.nii.gz' | PoCG.nii.gz' |
| 96 |  |  |  |  | 118.nii.gz' | PrCG.nii.gz' |
| 97 |  |  |  |  | 119.nii.gz' | PrCun.nii.gz' |
| 98 |  |  |  |  | 120.nii.gz' | Pu.nii.gz' |
| 99 |  |  |  |  | 121.nii.gz' | PuPV.nii.gz' |
| 100 |  |  |  |  | 122.nii.gz' | Pul.nii.gz' |
| 101 |  |  |  |  | 123.nii.gz' | RN.nii.gz' |
| 102 |  |  |  |  |  | Re.nii.gz' |
| 103 |  |  |  |  |  | ReG.nii.gz' |
| 104 |  |  |  |  |  | RoG.nii.gz' |
| 105 |  |  |  |  |  | SC.nii.gz' |
| 106 |  |  |  |  |  | SCG.nii.gz' |
| 107 |  |  |  |  |  | SEP.nii.gz' |
| 108 |  |  |  |  |  | SFG.nii.gz' |
| 109 |  |  |  |  |  | SIG.nii.gz' |
| 110 |  |  |  |  |  | SMG.nii.gz' |
| 111 |  |  |  |  |  | SN.nii.gz' |
| 112 |  |  |  |  |  | SOG.nii.gz' |
| 113 |  |  |  |  |  | SPL.nii.gz' |
| 114 |  |  |  |  |  | STG.nii.gz' |
| 115 |  |  |  |  |  | STH.nii.gz' |
| 116 |  |  |  |  |  | THM.nii.gz' |
| 117 |  |  |  |  |  | TI.nii.gz' |
| 118 |  |  |  |  |  | TP.nii.gz' |
| 119 |  |  |  |  |  | TTG.nii.gz' |
| 120 |  |  |  |  |  | VA.nii.gz' |
| 121 |  |  |  |  |  | VL.nii.gz' |
| 122 |  |  |  |  |  | VPL.nii.gz' |
| 123 |  |  |  |  |  | VPM.nii.gz' |
| 124 |  |  |  |  |  | ZI.nii.gz' |
| 125 |  |  |  |  |  | aLV.nii.gz' |
| 126 |  |  |  |  |  | ac.nii.gz' |
| 127 |  |  |  |  |  | bLV.nii.gz' |
| 128 |  |  |  |  |  | cc.nii.gz' |
| 129 |  |  |  |  |  | cec.nii.gz' |
| 130 |  |  |  |  |  | cpd.nii.gz' |
| 131 |  |  |  |  |  | fx.nii.gz' |
| 132 |  |  |  |  |  | iLV.nii.gz' |
| 133 |  |  |  |  |  | icp.nii.gz' |
| 134 |  |  |  |  |  | mcp.nii.gz' |
| 135 |  |  |  |  |  | mtt.nii.gz' |
| 136 |  |  |  |  |  | olt.nii.gz' |
| 137 |  |  |  |  |  | or.nii.gz' |
| 138 |  |  |  |  |  | ot.nii.gz' |
| 139 |  |  |  |  |  | pLV.nii.gz' |
| 140 |  |  |  |  |  | scp.nii.gz' |
| 141 |  |  |  |  |  | xLV.nii.gz' |

*The files of each atlas, ordered as they appear in Lead-DBS, are shaded red if they correspond to motor subnuclei, blue if sensory subnuclei, and green if other subnuclei. Unshaded files correspond to non-thalamic structures.
